# Massive Acute Spinal Subdural Hematoma Causing Sudden Onset Paraplegia in a Patient on Anticoagulation

**DOI:** 10.1155/2020/8898744

**Published:** 2020-11-13

**Authors:** Jacob Kosarchuk, Courtney Lewis, Martin H. Pham

**Affiliations:** ^1^Eastern Virginia Medical School, Norfolk, Virginia, USA; ^2^Department of Neurosurgery, University of California San Diego School of Medicine, San Diego, California, USA

## Abstract

Spinal subdural hematoma (SSDH) is a rare but known entity that can cause severe and irreversible motor, sensory, and autonomic dysfunction if not decompressed in a timely manner. We present here a 74-year-old female on anticoagulation who developed sudden onset back pain with rapidly progressive paraplegia. On neurologic exam, she was completely flaccid in the bilateral lower extremities with absent sensation from the umbilicus down. Imaging demonstrated a massive extra-axial spinal hematoma from T12 to S1 that initially was believed to be epidural in origin. She was taken emergently to the operating room for a T11-L5 decompressive laminectomy, and dural opening demonstrated a thick subdural clot encasing the conus and cauda equina confirming the subdural pathology. Despite decompression and partial evacuation of the subdural hematoma, she did not recover neurologic function.

## 1. Introduction

Spinal subdural hematoma (SSDH) is a rare but known entity that can cause severe and irreversible sensorimotor and autonomic dysfunction if not decompressed in a timely manner [1–4]. We report here a case of a massive thoracolumbar SSDH in the setting of therapeutic anticoagulation causing acute-onset paralysis.

## 2. Literature Search

A literature search was performed to determine if SSDHs of this size and severity of symptoms were reported elsewhere. We searched PubMed using the terms “spinal subdural hematoma,” “massive spinal subdural hematoma,” “acute spinal subdural hematoma,” with the qualified “AND anticoagulation,” “AND warfarin,” “AND heparin,” “AND aspirin,” “AND apixaban,” “AND rivaroxaban,” “AND dabigatran,” with article-type filters for case reports, clinical studies, clinical trials, comparative studies, observational studies, reviews, and systematic reviews. We also performed reviews of citations within articles we found. Our initial search yielded 1,066 articles. After filtering for atraumatic or nonprocedural SSDH, we found 202 articles. Those that did not reference an anticoagulant in the title or body of the article were excluded, leaving a total of 24 articles.

## 3. Case Report

**Figure 1:**
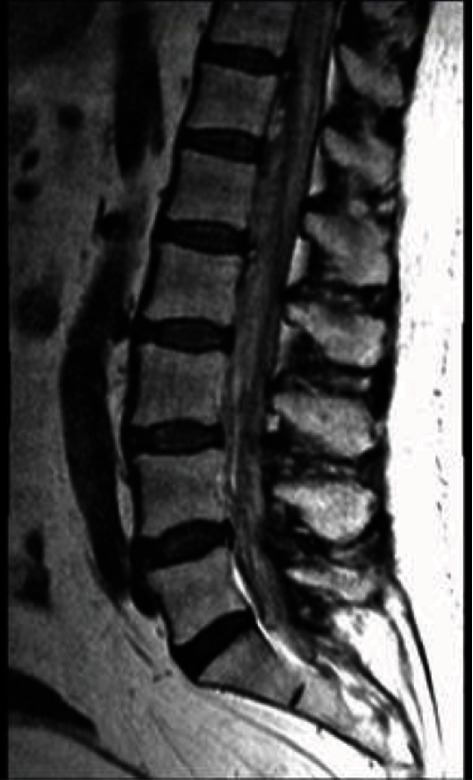
T2-weighted MRI of the lumbar spine demonstrating ventral hypointensity relative to CSF, compressing the conus and cauda equina.

We present here a 74-year-old female on anticoagulation who developed sudden onset back pain and rapidly progressive flaccid paraplegia. On neurologic exam, she was completely flaccid in the bilateral lower extremities with absent sensation from the umbilicus down. Magnetic resonance imaging (MRI) demonstrated a massive ventral spinal subdural hematoma from T12 to S1 (Figure 1). Due to these findings, she was taken emergently to the operating room for decompression and evacuation.

## 4. Operation

**Figure 2:**
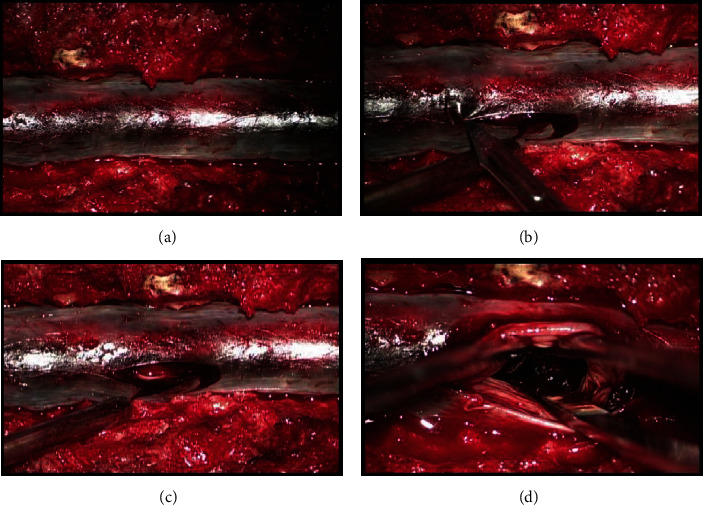
Operative microscope views of the dorsal thoracolumbar spinal dura. Note the mottled appearance of the dural sac (a). Opening of the dural sac (b, c) demonstrates thick clotted blood intradurally. The massive volume of the subdural blood has filled the intradural space and obliterated all egress of cerebrospinal fluid (d).

A T11-L5 laminectomy was performed for complete epidural decompression. Dural opening demonstrated a thick subdural clot encasing the conus and cauda equina (Figure 2). A partial evacuation was performed focusing on the proximal hematoma at the spinal cord and conus; the rest of the clot distal at the cauda equina was partially removed with a combination of direct evacuation and irrigation due to the difficult consistency of the clot encasing the cauda equina roots.

## 5. Postoperative Course

Postoperatively, she did not have any recovery of strength or sensation. She was monitored as an inpatient for 3 days and subsequently discharged to an acute rehabilitation center. At six months follow-up, there has been no recovery of neurologic function.

## 6. Literature Review Results

Our review of the literature resulted in 202 articles discussing atraumatic and nonprocedural iatrogenic SSDH. They included case reports, reviews on management of SSDH, and reviews on imaging diagnosis of SSDH. We found 24 case reports on SSDH in patients who had been anticoagulated (Table 1). There was an even distribution of male and female patients (12 males and 12 females) found in this literature review, and the average age was 63.06 years with a range of 38 to 80 years old (Table 2). The majority of patients in the series had atrial fibrillation as a comorbidity (15/24), with others including stroke (2/24), cardiovascular and disease (3/24), venous thromboembolism (2/24), cardiac valve replacement (2/24), and other (1/24). 2/24 were on low molecular weight heparin (LMWH), 4/24 were on aspirin (in combination therapies), 10/24 were on warfarin, 2/24 were on clopidogrel, 1/24 was on ticlodipine, 3/24 were on apixaban, 4/24 were on rivaroxaban, and 1/24 was on dabigatran. 13/24 patients had multilevel or diffuse SSDH, 9/24 were confined to the thoracic region and 1/24 to the lumbar region, and none had purely cervical or sacral SSDH. 19/24 had no associated subarachnoid hemorrhage (SAH), 4/24 had definite SAH, and 1/24 had indeterminate SAH. 5/24 patients did not improve, 13/24 partially improved, 5/24 fully recovered, and 1 patient died of a cardiac arrest. 16/24 patients received operative intervention, and 8/24 received conservative treatment (including the patient that died). Of the patients that did not improve, all 5 underwent surgical intervention. 10/13 in the partial improvement had surgery compared to 3/13 who were managed conservatively. 4/5 patients who fully recovered were managed conservatively, and 1/5 was operated on.

## 7. Discussion

We present the case of a massive spinal subdural hematoma in an elderly female on anticoagulation causing severe back pain and rapid-onset paraplegia. SSDH as an entity has been previously described, including in association with anticoagulation. We report here a unique case of a massive thoracolumbar SSDH that initially was believed on radiological review to be a ventral epidural hematoma in origin.

Spontaneous spinal subdural hematoma (sSSDH) is a rare cause of back pain, paraplegia, and cauda equina syndrome and should be considered in a patient who is on anticoagulation, and no other precipitating events are identified [3]. The average age of patients in this case series was 63.06 years (note—one study simply reported the age as “middle aged”), which is similar to a recent study by Pereira et al. but differs from other older studies [3, 4]. We found an even distribution of males (50%) and females (50%) in this case series, which is similar to previously reported rates [1, 4]. The majority of patients in this series were on warfarin [5–14], which could be due to a higher rate or longer duration of warfarin use compared to newer novel oral anticoagulants (NOACs) and not necessarily due to the agent itself, though studies have shown lower rates of (unspecified) major bleeding events with NOACs [15–23]. We identified fewer patients on other agents (including antiplatelet therapies) that developed SSDH [14, 24–28].

SSDHs are often associated with coagulopathies (iatrogenic or related to impaired innate hemostasis mechanisms) and procedural iatrogenic causes, though there is still a significant amount of SSDHs secondary to arteriovenous malformations, trauma, and idiopathic causes [3, 4, 6]. The pathophysiology of spontaneous SSDH is still unclear but is theorized to be caused by bleeding within the subdural space itself or as an extension of a subarachnoid bleed into the subdural space after an increase in intrathoracic or intra-abdominal pressure [1, 3]. Indeed, there have been cases of concomitant SAH and SSDH [7, 9, 14, 18, 21]. Important prognostic factors include neurologic status at presentation, presence of coagulopathy, performance of lumbar puncture, and associated diseases [4]. Interestingly, extension of hematoma, surgery, and presence of SAH were not found to be significant predictors of outcome.

MRI is considered the gold standard in the diagnosis of SSDH, but digital subtraction angiography may be useful if spinal AVM is suspected [2]. SSDH can be managed conservatively with medical management (often including steroids), percutaneous drainage, and surgical evacuation [3, 29]. Though the literature suggests that conservative management results in better outcomes, this may be related to bias in choosing patients with less severe symptoms for medical management whereas more impaired patients are selected for surgical intervention [4].

## 8. Conclusion

SSDH is a rare but serious cause of rapid-onset back pain, sensorimotor, and autonomic deficits, and in some cases, mortality. It is often associated with iatrogenic causes including anticoagulation (as in this case) but in some instances may be idiopathic. MRI is the gold standard for diagnosis. Patients with mild or moderate symptoms can be managed conservatively, but urgent surgical decompression and clot evacuation are warranted in patients with severe symptoms to prevent permanent neurologic injury or death.

## Figures and Tables

**Table 1:** Results and summary of the case reports identified in the literature search.

Patient characteristics								
Sex	Male	Female						
	12	12						
Age	Average	Min	Max					
	63.06	38	80					
Comorbidities	A-fib	Stroke	CVD	PE/DVT	Valve replacement	Other		
	15	2	3	2	2	1		
Agent used	LMWH	ASA	Warfarin	Clopidogrel	Ticlodipine	Apixaban	Rivaroxaban	Dabigatran
	2	4	10	2	1	3	4	1
Location of bleed	Cervical	Thoracic	Lumbar	Sacral	Multilevel			
	0	9	1	0	13			
Associated SAH	Yes	No	Indeterminate					
	19	4	1					
Intervention	Operative	Nonoperative						
	16	8						
Recovery	No improvement	Some improvement	Full recovery	Death				
	5	13	5	1				
Recovery vs. intervention	No improvement		Some improvement		Full recovery		Death	
	Op	Non-Op	Op	Non-Op	Op	Non-Op	Op	Non-Op
	5	0	10	3	1	4	0	1

**Table 2:** Characteristics of patients in the literature review.

Author and year	Age (years)	Sex	Location	Presenting symptoms	Anticoagulant/antiplatelet	Risk factors	SAH	Treatment	Outcome
Miller 2004	67	M	T11-S1	Progressive loss of sensation, bilateral leg weakness, back pain, urinary retention	Warfarin	Atrial fibrillation	None	T10-L2 laminectomy	No recovery
Cha 2005	72	F	T3-T6	Bilateral lower extremity paraplegia, sensory loss, urinary retention, back pain	LMWH, ASA	Laryngeal cancer, NSTEMI	None	T3-T5 laminectomy	Minimal recovery
Chau 2008	52	M	C7-L1	Bilateral lower limb weakness, decreased lower extremity sensation, bowel and bladder dysfunction	LMWH	Salmonella typhi, reactive arthritis, type II diabetes	None	T11-T12 laminectomy	Partial recovery
Badge 2009	78	F	T3-T12	Nausea, giddiness, headache, back pain, progressive L lower extremity weakness and ataxia	Warfarin	Atrial fibrillation	None	T5 laminectomy	Improved
Mete 2010	42	M	T6-L5	Sudden headache and backache, agitation, paraparesis, meningismus	Warfarin	Cardiac pacemaker, h/o cardiac bypass	Yes	Conservative	Death (cardiac arrest)
Payer 2010	59	M	T2-T9	Left-dominant paraparesis below T8, weakness, sphincter dysfunction	ASA, clopidogrel	Cardiac stenting	None	Conservative	Full strength recovery at 1 year, improvement in ataxia, paresthesia, urge incontinence
Wang 2012	67	F	L4-S1	Back pain, bilateral radiating leg pain, bifrontotemporal headache, bilateral lower extremity weakness and numbness	ASA, clopidogrel	Atrial fibrillation, concomitant SDH	None	Conservative (SSDH), burr hole craniotomy (SDH)	Full recovery at 1 year
Bruce-Brand 2013	76	M	L1-L4	Sudden onset severe low back pain, lower extremity weakness	Warfarin	Atrial fibrillation, type II diabetes, HTN, dyslipidemia	None	T12-L4 laminectomy	Partial recovery at 6 months
Castillo 2015	69	M	T3-conus	Back pain, bilateral lower extremity paraplegia, bowel and bladder dysfunction	Rivaroxaban	Atrial fibrillation	None	Cervical and lumbar drains	No recovery at 6 months
Dargazanli 2015	72	M	T6-T8	Interscapular back pain, rapidly progressive bilateral lower extremity paraplegia, decreased lower extremity sensation to pain and temperature L > R	Rivaroxaban	Atrial fibrillation	None	T6-T8 laminectomy	No improvement at 6 months
Frioui 2015	65	F	T12-L1	Back pain, paraparesis, urinary retention	Unclear anticoagulant	Atrial fibrillation, HTN	None	Conservative	Neurological recovery observed at 24 h. Persistent urinary retention at 1 year
Jung 2015	53	F	C7-T6	Sudden onset headache, nausea, vomiting, meningismus, bilateral lower extremity sensory loss and weakness, back pain	Warfarin	Aortic valve replacement	Yes	C7-T6 laminectomy	Improvement in anal tone and bladder function
Zaarour 2015	58	M	C7-T2	Interscapular back pain, progressive bilateral lower extremity weakness and numbness	Rivaroxaban	Atrial fibrillation, type II diabetes, HTN, dyslipidemia, recent THA with spinal anesthesia	None	Initially conservative, C7 corpectomy at HD4	Improved
Siasios 2016	38	F	Diffuse	Severe neck and back pain	Warfarin	Recent C-section, PE	None	Conservative	Full recovery at discharge
Wolfe 2017	67	M	Cervicothoracic	Left lower extremity weakness, urinary retention	Dabigatran	Atrial fibrillation, endocarditis, PCKD s/p transplant, BPH, OSA, melanoma	Yes	Conservative	Full recovery in 2 days
Akiyama 2017	71	M	T7-S1	2 weeks history bilateral lower leg pain, dysesthesia, paraparesis, urinary disturbance, and fever	Ticlodipine	L cerebral infarct, unruptured L MCA aneurysms	None	L3-L4 laminectomy	Improved
Bunevicius 2017	68	M	T2-T6	Left-sided chest and back pain, L leg weakness, and R leg numbness	Warfarin	Atrial fibrillation	None	T3-T6 hemilaminectomy	Partial improvement
Bang 2018	Middle aged	F	Thoracolumbar	Lower extremity weakness after assault^∗∗^	Rivaroxaban	Atrial flutter	None	Laminectomy	No improvement
Colell 2018	75	F	Diffuse	Bilateral lower extremity weakness L > R, decreased sensation	Apixaban	Atrial fibrillation, HTN	None	Laminectomy	Improved at 6 months
Girithari 2018	57	F	T4-T8	Back pain, headache, vomiting, bilateral lower extremity weakness, urinary retention	Warfarin	DVT	None	Laminectomy	No improvement at 6 months
Mchaourab 2018	68	M	T1-T5	Back pain, urinary retention, headache, neck stiffness, vomiting, bilateral limb weakness, and ataxia	Apixaban	Atrial fibrillation	Possible	Conservative	Partial improvement
Arain 2019	80	F	T4-T9	Bilateral lower extremity paraplegia, incontinence	Warfarin	Atrial fibrillation	None	T3-T11 laminectomy	Partial improvement
Ardebol 2019	67	F	T4-T7	Back pain, progressive bilateral lower extremity paraplegia, bowel and bladder dysfunction	Apixaban	Atrial fibrillation	None	T4-T7 laminectomy	Full recovery at 1 year
Weiner 2019	42	F	L1-S1	Vaginal pain, back pain radiating down the right leg, left leg weakness, frontal headache	Warfarin, ASA	Rheumatic heart disease, mechanical mitral valve, CVA, concomitant SDH, concomitant arachnoiditis	Yes	Craniotomy for SDH, conservative for SSDH	Full recovery at 1 month

## Data Availability

The imaging and photographic data used to support the findings of this study are included within the article.
